# Milk Consumption and Cardiovascular Risk Factors in Older Chinese: The Guangzhou Biobank Cohort Study

**DOI:** 10.1371/journal.pone.0084813

**Published:** 2014-01-08

**Authors:** Yangbo Sun, Chaoqiang Jiang, Kar Keung Cheng, Weisen Zhang, Gabriel M. Leung, Tai Hing Lam, C. Mary Schooling

**Affiliations:** 1 Lifestyle and Lifecourse Epidemiology Group, School of Public Health, Li Ka Shing Faculty of Medicine, The University of Hong Kong, SAR, China; 2 Guangzhou Number 12 Hospital, Guangzhou, China; 3 Department of Public Health and Epidemiology, University of Birmingham, Birmingham, United Kingdom; 4 School of Public Health, CUNY, New York, New York, United States of America; Medical University Innsbruck, Austria

## Abstract

**Background:**

Dairy products consumption is increasingly common globally. Most of the evidence concerning dairy products comes from observational studies in western populations which are inevitably open to confounding. To triangulate the evidence concerning dairy products, we examined the associations of whole cow's milk consumption with cardiovascular risk factors in a non-Western setting with a different pattern of milk consumption and cardiovascular diseases from Western populations.

**Methods:**

We used multivariable censored linear or logistic regression to examine cross-sectionally the adjusted associations of whole cow's milk consumption (none (n = 14892), 1–3/week (n = 2689) and 3+/week (n = 2754)) with cardiovascular risk factors in Chinese (≥50 years) in the Guangzhou Biobank Cohort Study.

**Results:**

Whole cow's milk consumption was negatively associated with systolic blood pressure (3+/week compared to none −2.56 mmHg, 95% confidence interval (CI) −3.63 to −1.49), diastolic blood pressure (−1.32 mmHg, 95% CI −1.87 to −0.77) and triglycerides (−0.06 mmol/L, 95% CI −0.11 to −0.002), but was positively associated with HDL-cholesterol (0.02 mmol/L,95% CI 0.01 to 0.04) and fasting glucose (0.08 mmol/L, 95% CI 0.01 to 0.16) adjusted for age, sex, phase of study, socio-economic position, lifestyle (smoking, alcohol use and physical activity) and adiposity, but had no obvious association with LDL-cholesterol or the presence of diabetes.

**Conclusions:**

Whole cow's milk consumption had heterogeneous associations with cardiovascular risk factors. Higher whole cow's milk consumption was associated with lower levels of specific cardiovascular risk factors which might suggest risk factor specific biological pathways with different relations to blood pressure and lipids than glucose.

## Introduction

Cardiovascular disease (CVD) is the leading cause of death worldwide[1]. According to the World Health Organization, by 2030, almost 24 million people will die from CVD, mainly from heart disease and stroke[1]. Observational studies in western settings suggest cow's milk consumption influences CVD and its risk factors[2], including diabetes[3]. Meta-analyses of prospective cohort studies found a modest inverse association of total milk intake with CVD in western settings, and possibly diabetes[3]. However, when studies were restricted to whole milk, prospective cohort studies show no association or whole milk positively associated with CVD and diabetes[3]–[6]._ENREF_5 _ENREF_6_ENREF_9Inevitably with observational studies of a socially and culturally patterned behavior it is uncertain whether these associations are due to the biological properties of cow's milk or to other attributes of cow's milk drinkers, because in western settings higher socio-economic position (SEP) is usually associated with higher consumption of low-fat milk and cheese, lower consumption of full fat milk[7]–[9]_ENREF_6 and lower morbidity and mortality from many chronic diseases including CVD and diabetes[10]. As such the observed associations of cow's milk consumption with chronic diseases might be confounded by SEP. Nevertheless cow's milk consumption features in many food guidelines[11], and is becoming increasingly common globally with economic development[12].

To our knowledge, no randomized controlled trial (RCT) or Mendelian randomization study has reported the effect of whole cow's milk consumption on CVD. Three small short-term RCTs have reported the effect of whole cow's milk consumption on some CVD risk factors. Among 45 young adults from Spain whole cow's milk increased systolic blood pressure (SBP) but had no effect on diastolic blood pressure (DBP)[13]. Among 32 boys from South Africa whole cow's milk increased high density lipoprotein (HDL)-cholesterol, decreased triglycerides but had no effect on low density lipoprotein (LDL)-cholesterol[14]. Among 30 young women from Japan whole cow's milk increased HDL-cholesterol but had no effect on triglycerides[15]. A small Mendelian randomization study found lactase persistence, which enables people to digest lactose (milk sugar), and may reflect higher cow's milk consumption, non-significantly positively associated with CVD risk factors, such as SBP or DBP, lower HDL-cholesterol, triglycerides and fasting plasma glucose[16], compared to lactase non-persistence.

Given the limited experimental evidence concerning whole cow's milk consumption, evidence from the majority of the global population in non-western settings with potentially less or different confounding may help identify whether the observed associations of cow's milk consumption with CVD risk factors are biologically based or contextually specific results of unmeasured confounding. Moreover, the range of cow's milk consumption is also wider in non-western settings, where it is normal and common to consume little or no cow's milk. Clarifying the role of cow's milk in such settings now during the ‘window of opportunity’ before cow's milk becomes an established part of the diet may also inform key public health policies. Prospective cohort studies from the developed East Asian setting of Japan found cow's milk consumption inversely associated with CVD[17], [18], but not obviously associated with diabetes[19]. The only study, to date, from China, found whole cow's milk consumption negatively associated with type 2 diabetes[20]. Here, we examined the associations of whole cow's milk consumption with CVD risk factors in a large study of older adults (50+ years) from the economically developing non-western setting of Guangzhou in Southern China, using the Guangzhou Biobank Cohort Study. We also examined whether these associations varied by sex or age.

## Methods

### Ethics statement

The Guangzhou Medical Ethics Committee of the Chinese Medical Association approved the study and all participants gave written, informed consent before participation.

### Materials

The Guangzhou Biobank Cohort Study (GBCS) is a collaboration between the Guangzhou No. 12 Hospital and the Universities of Hong Kong and Birmingham and has been described in detail elsewhere[21]. Participants were recruited from “The Guangzhou Health and Happiness Association for the Respectable Elders”, a community social and welfare association unofficially aligned with the municipal government where membership is open to anyone aged 50 years and over for a nominal monthly fee of 4 Yuan (50 US cents). Participants were recruited into the study in three recruitment phases: phase 1 took place from September 2003 to September 2004, phase 2 from April 2005 to May 2006, and phase 3 from September 2006 to January 2008, with 10413, 9998 and 10088 recruited participants respectively. About 7% of permanent Guangzhou residents aged 50 years or older are members of this association, of whom 33% enrolled for phases1, 2 and 3 recruitment. They were included if they were capable of consenting, ambulatory, and not receiving treatment modalities which if omitted might result in immediate life-threatening risk, such as chemotherapy or radiotherapy for cancer, or dialysis for renal failure. Participants underwent a half-day detailed medical interview, including lifestyle and other risk factors, disease history and physical examination during each recruitment phase.

In brief, seated blood pressure was recorded as the average of the last 2 of 3 measurements, using the Omron 705CP sphygmomanometer (Omron Corp, Kyoto, Japan). Standing height without shoes was measured to the nearest 0.1 centimeter. Weight (with participants dressed in light clothing) was measured to the nearest 0.1 kilogram. We measured hip circumference at the greatest circumference around the buttocks below the iliac crest and waist circumference horizontally around the smallest circumference between the ribs and iliac crest, or at the level of the naval for obese participants. Levels of HDL-cholesterol, LDL-cholesterol, triglycerides and plasma glucose were determined using the Shimadzu CL-8000 Clinical Chemical Analyzer (Shimadzu Corp, Kyoto, Japan) in the hospital laboratory.

### Exposures

Dairy products was obtained from a validated food frequency questionnaire used in phases 1 and 2, but not phase 3 where a different dietary instrument was used[21], including whole cow's milk, skimmed milk, chocolate milk, dried whole milk, dried skimmed milk, sweetened condensed milk, ice cream and milk shake. Participants were asked the portions and frequency of each dairy product consumed in the past week. Whole cow's milk was consumed by 27% of the participants while less than 8% ever consumed other types of dairy products. Moreover, whole cow's milk contains the full nutrition of cow's milk providing an overall effect of the nutrition contained in cow's milk on human health. As such whole cow's milk was chosen as the main exposure. Milk consumption was reported as portions per week and categorized into never, 1–3 portions (1 portion = 250 ml) per week and >3 portions per week, based on the frequency of consumption and the usual amount per occasion.

### Outcomes

Ten biological outcomes were examined: SBP, DBP, pulse pressure, HDL-cholesterol, LDL-cholesterol, triglycerides, fasting plasma glucose, body mass index (BMI), waist-hip ratio (WHR) and the presence of diabetes (defined as previous diagnosis, fasting plasma glucose ≥7.0 mmol/L, or on antidiabetic medication). These outcomes were chosen to reflect the biological risk factors in the Framingham equation[22], a risk prediction equation for ischemic heart disease (IHD), and the Diabetes Prediction Model, another clinical risk-scoring system[23]. In younger people, SBP and DBP track together. In older people, however, whereas SBP continues to rise, DBP falls, and the difference between the two (pulse pressure) contributes to IHD risk [24].

### Covariates

We included age, sex, study-phase, socio-economic position (SEP), lifestyle, BMI and WHR as potential confounders in the analysis. SEP was measured at four life stages: childhood SEP (father's occupation), early adulthood SEP (education), late SEP (longest-held education) and current SEP (annual personal income in yuan). Occupation was categorized as manual (agricultural work, factory work, or sales and services), non-manual (administrative/managerial, professional/technical, or military/police) or others (housewife/husband or retired). Lifestyle included smoking, alcohol use and physical activity. Smoking was classified as never smoked, ex-smoker, and current smoker. Alcohol use was classified as never, less than once per week, 1–4 times per week, 5 or more times per week, and ex-drinker. Physical activity was categorized as inactive, minimally active and HEPA (Health Enhancing Physical Activity) active. The full range of values of covariates is shown in Table 1.

**Table 1:** Characteristics by Whole Cow's Milk Consumption in Older Chinese (5853 men and 14,482 women) in Phases 1 and 2 of the Guangzhou Biobank Cohort Study, 2003–2006.

	Whole cow's milk consumption (aportions/week)							
	Men				Women			
	0	1–3	>3	c *P* value	0	1–3	>3	c *P* value
N	4360	721	772		10532	1968	1982	
Age group (%)								
50–54	5.32	12.34	6.61		14.41	25.71	18.62	
55–59	18.37	18.86	17.23		27.24	31.20	26.79	
60–64	25.46	26.77	24.61		21.61	18.19	20.74	
65–69	27.50	25.24	28.11		22.32	16.72	21.19	
70–74	19.68	14.56	18.91		11.86	7.37	11.05	
75–79	3.19	1.94	3.89		2.23	0.66	1.51	
≥80	0.48	0.28	0.65	**<0.001**	0.32	0.15	0.10	**<0.001**
Education (%)								
Less than primary school	3.21	1.94	0.91		16.53	7.12	6.87	
Primary school	31.01	21.50	16.71		39.74	32.89	30.39	
Junior middle school	28.72	30.93	26.55		23.25	29.84	27.26	
Senior middle school	21.46	24.27	27.72		15.78	23.34	25.69	
Junior college	8.26	10.68	15.16		3.12	4.63	5.86	
College or above	7.32	10.68	12.95	**<0.001**	1.59	2.19	3.94	**<0.001**
Job type (%)								
Manual	49.29	43.83	36.27		69.32	59.81	53.48	
Non-manual	39.68	35.37	47.80		18.56	17.94	25.48	
Other	11.03	20.80	15.93	**<0.001**	12.12	22.26	21.04	**<0.001**
Father's occupation (%)								
Manual	47.13	48.54	45.60		45.37	46.95	40.21	
Non-manual	11.33	13.04	15.03		11.53	12.09	14.63	
Other	41.54	38.42	39.38	**0.029**	43.11	40.96	45.16	**<0.001**
^b^Income group (%)								
<10000 yuan (∼US$1250)	23.39	19.97	16.78		47.94	39.45	36.15	
10000 to 15000 yuan	45.45	45.30	43.89		41.50	48.47	48.42	
≥15000 yuan	31.17	34.73	39.33	**<0.001**	10.56	12.08	15.43	**<0.001**
Smoking status (%)								
Never	38.75	44.94	48.19		95.42	97.36	97.28	
Ex-smoker	29.94	26.35	31.48		2.08	1.27	1.31	
Current smoker	31.31	28.71	20.34	**<0.001**	2.50	1.37	1.41	**<0.001**
Alcohol use (%)								
Never	58.75	64.02	65.55		89.59	89.85	88.88	
<1/month	14.57	16.43	14.42		6.14	7.36	6.62	
<1/week	4.17	5.24	4.41		1.04	1.16	1.76	
1–4/week	8.86	6.94	7.48		1.17	0.63	1.45	
>5/week	10.33	7.08	6.81		1.38	0.89	1.09	
Ex-drinker	3.31	0.28	1.34	**<0.001**	0.68	0.11	0.21	**<0.001**
Physical activity (IPAQ) (%)								
Inactive	8.35	9.71	7.90		7.82	10.47	7.62	
Minimally active	48.35	53.81	50.52		45.20	52.39	49.45	
HEPA	43.30	36.48	41.58	**0.013**	46.98	37.14	42.94	**<0.001**
Body mass index	23.54	23.46	23.39	0.46	23.94	23.81	23.54	**<0.001**
Waist-hip ratio	0.901	0.899	0.897	0.18	0.859	0.847	0.845	**<0.001**

Abbreviations: IPAQ, International Physical Activity Questionnaire; HEPA, health-enhancing physical activity, i.e., vigorous activity at least 3 days a week that corresponds to a minimum of 1500 metabolic equivalent (MET) minutes per week, or activity 7 days of the week that corresponds to at least 3000 MET minutes per week.

^a^ 1 portion = 250 ml; ^b^US$1 = 8 yuan (according to exchange rate from 2003 to 2006).

^c^ *P* value from chi-square test for categorical variables and from one-way analysis of variants (ANOVA) for continuous variables, 2 sided; bold values indicate *P*<0.05.

### Statistical analysis

We did a cross-sectional study of the baseline survey to examine the associations of exposures with outcomes. The associations of potential confounders with milk consumption were examined using chi-square tests for categorical variables and one-way analysis of variance (ANOVA) for continuous variables. Multivariable censored linear regression was used to assess the adjusted associations of whole cow's milk with blood pressure, lipids and fasting plasma glucose because some people were taking medications for hypertension (n = 4738), hyperlipidemia (n = 1393) or diabetes (n = 1662). These models censored the outcome for those on medication at the observed value so that the true measurement for blood pressure, LDL-cholesterol, triglycerides and fasting plasma glucose was assumed to be that observed or higher, while the true measurement for HDL-cholesterol was assumed to be that observed or lower. Multivariable linear regression was used to assess the adjusted associations of whole cow's milk consumption with BMI and WHR, and multivariable logistic regression was used to assess the adjusted association of whole cow's milk consumption with diabetes.

### Sensitivity analysis

Given whole milk consumption may be modified as part of lifestyle changes with CVD, we repeated the analysis excluding people with existing CVD (defined as self-reported CVD, or use of CVD medication) or diabetes defined as self-reported diabetes, or use of antidiabetic medication).

We included potential confounders sequentially in the models. Model 1 adjusted for sex, age (in 5-year age groups) and phase. Model 2 additionally adjusted for socio-economic position (SEP) (father's occupation, education, longest-held occupation and personal income) and lifestyle (smoking, alcohol use and physical activity), as categorized in Table 1. The causal role of milk consumption in adiposity is unclear, so model 3 additionally adjusted for BMI and WHR. We examined whether CVD risk factors had different associations with whole milk consumption by sex or age from the heterogeneity across subgroups and the p-values of the relevant interaction terms in models including interactions with other confounders to avoid confounding by these other interactions.

Information on exposure (whole cow's milk) and most potential confounders was missing for less than 5% apart from longest-held occupation (14.2%) and father's occupation (42.5%). Initial analysis showed that using dummy categories for the 2885 participants with missing occupation and for the 8633 participants with missing father's occupation produced similar results to multiple imputations of all missing exposures and potential confounders[25] from a flexible additive regression model with predictive mean matching incorporating all exposures, potential confounders and outcomes with confidence intervals adjusted for the missing data uncertainty. So, we present results using dummy categories for the missing occupation and missing father's occupation.

## Results

Of the original 20,335 participants in phase 1 (10,407) and phase 2 (9,928) of GBCS, less than 120 had missing information on any CVD risk factor, so we used all available observations for each outcome. There were more women (14,482) than men (5853), and the women were younger (mean age 61.9 (standard deviation (SD) 6.7) than the men (mean age 64.7 (SD 6.3)). Age ranged from 50 to 95 years, but only 527 participants were older than 75 years.

Table 1 shows the associations of whole cow's milk consumption with potential confounders. Among all the participants, 14,892 never drank whole cow's milk, 2689 drank 1–3 portions per week and 2754 drank >3 portions per week. For men and women, higher whole cow's milk consumption was associated with higher education, non-manual occupation (of self and father) and higher personal income, but the pattern of associations with age, smoking status, alcohol use and physical activity was unclear. For women, but not men, higher whole cow's milk consumption was associated lower BMI and WHR.

Table 2 shows the distribution of CVD risk factors and unadjusted associations of whole cow's milk consumption with CVD risk factors. Higher whole milk consumption was associated with lower SBP, lower DBP, higher HDL-cholesterol, lower LDL-cholesterol, lower triglycerides, lower BMI and lower WHR, but had no obvious association with pulse pressure, fasting plasma glucose, or diabetes.

**Table 2:** Unadjusted Associations of Whole Cow's Milk Consumption with Cardiovascular Risk Factors in Older Chinese (5853 men and 14,482 women) in Phases 1 and 2 of the Guangzhou Biobank Cohort Study, 2003–2006.

	Mean and standard deviation		Whole cow's milk consumption (bportions/week)				
			0	1–3		>3	
aCVD risk factor	Men	Women		ccoefficient	95% CI	coefficient	95% CI
Systolic blood	133.7	130.2	reference	**−4.73**	−5.84, −3.62	−**4.79**	−5.89, −3.69
pressure (mm Hg)	±21.6	±22.3					
Diastolic blood	76.5	72.9	reference	−**1.90**	−2.46, −1.34	−**2.43**	−2.99, −1.88
pressure (mm Hg)	±11.5	±11.1					
Pulse pressure	57.2	57.4	reference	−**2.95**	−3.72, −2.19	−**2.43**	−3.19, −1.67
(mm Hg)	±14.7	±15.7					
HDL-cholesterol	1.53	1.73	reference	**0.02**	0.01, 0.04	**0.03**	0.01, 0.05
(mmol/L)	±0.39	±0.40					
LDL-cholesterol	2.99	3.26	reference	**0.13**	0.10, 0.16	**0.10**	0.07, 0.13
(mmol/L)	±0.64	±0.70					
Triglycerides	1.58	1.66	reference	−**0.08**	−0.13, −0.03	−**0.09**	−0.14, −0.04
(mmol/L)	±1.21	±1.25					
Fasting plasma	5.73	5.79	reference	−**0.20**	−0.28, −0.13	−0.06	−0.13, 0.02
glucose (mmol/L)	±1.58	±1.78					
Body mass index	23.5	23.9	reference	−0.11	−0.25, 0.03	−**0.32**	−0.46, −0.19
	±3.2	±3.4					
Waist-hip ratio	0.90	0.86	reference	−**0.01**	−0.013, −0.007	−**0.01**	−0.014, −0.009
	±0.06	±0.06					
	Prevalence			OR	95% CI	OR	95% CI
Diabetes	12.31%	13.86%	reference	**0.87**	0.76, 0.98	1.02	0.91, 1.15

a Censored regression was used for systolic blood pressure, diastolic blood pressure, pulse pressure, HDL, LDL, triglyceride and fasting plasma glucose; linear regression was used for body mass index and waist-hip ratio; logistic regression was used for diabetes.

b 1 portion = 250 ml.

c Coefficient means changes in risk factors; bold values indicate *P*<0.05.

Table 3 shows the adjusted associations of whole cow's milk consumption with CVD risk factors. Whole cow's milk consumption was associated with lower SBP, lower DBP, lower pulse pressure, higher HDL-cholesterol, lower triglycerides, lower BMI and lower WHR, but had no obvious association with LDL-cholesterol, fasting plasma glucose or diabetes. Adjustment for confounders (model 2 and model 3 compared with model 1) somewhat attenuated these associations. Whole milk consumption was only associated with higher fasting plasma glucose after adjustment for adiposity. Results using multiple imputation were similar, as shown in the Table S1 in File S1. Results without adjusting for medication usage were similar (data not shown). When people with previous CVD or diabetes were excluded from the analysis, the results were similar except that the negative association of whole milk consumption with triglycerides was somewhat attenuated, and whole milk consumption was no longer associated with fasting plasma glucose, as shown in Table S2 in File S1. Results remained similar after adjusting for soy milk consumption (data not shown).

**Table 3:** Adjusted Associations of Whole Cow's Milk Consumption with CVD Risk Factors in Older Chinese (5853 men and 14,482 women) in Phases 1 and 2 of the Guangzhou Biobank Cohort Study, 2003–2006.

				Whole cow's milk consumption (cportions/week)					*P*-value for trend
		N		0	1–3		>3		
aCVD risk factor	bModel	Men	Women		dcoefficient	95% CI	coefficient	95% CI	
Systolic blood	1	5826	14431	reference	−**1.59**	−2.68, −0.50	−**3.57**	−4.64, −2.50	**<0.001**
pressure (mm Hg)	2	5445	13422	reference	−**1.33**	−2.47, −0.21	−**3.28**	−4.39, −2.17	**<0.001**
	3	5424	13369	reference	−**1.10**	−2.20, −0.02	−**2.56**	−3.63, −1.49	**<0.001**
Diastolic blood	1	5825	14430	reference	−**0.86**	−1.43, −0.30	−**1.83**	−2.38, −1.28	**<0.001**
pressure (mm Hg)	2	5444	13421	reference	−**0.74**	−1.33, −0.16	−**1.70**	−2.28, −1.13	**<0.001**
	3	5423	13368	reference	−**0.62**	−1.18, −0.06	−**1.32**	−1.87, −0.77	**<0.001**
Pulse pressure	1	5822	14425	reference	−0.72	−1.44, 0.01	−**1.76**	−2.47, −1.05	**<0.001**
(mm Hg)	2	5441	13416	reference	−0.65	−1.40, 0.10	−**1.68**	−2.42, −0.94	**<0.001**
	3	5420	13363	reference	−0.54	−1.28, 0.20	−**1.31**	−2.04, −0.58	**<0.001**
HDL-cholesterol	1	5826	14414	reference	**0.03**	0.01, 0.05	**0.03**	0.02, 0.05	**<0.001**
(mmol/L)	2	5447	13407	reference	**0.03**	0.01, 0.05	**0.03**	0.01, 0.05	**<0.001**
	3	5414	13334	reference	**0.03**	0.01, 0.04	**0.02**	0.01, 0.04	**0.002**
LDL-cholesterol	1	5812	14370	reference	0.02	−0.01, 0.05	0.03	−0.003, 0.05	**0.042**
(mmol/L)	2	5433	13364	reference	0.02	−0.01, 0.05	0.02	−0.01, 0.05	0.17
	3	5400	13291	reference	0.02	−0.01, 0.05	0.02	−0.01, 0.05	0.08
Triglycerides	1	5827	14419	reference	−**0.09**	−0.14, −0.03	−**0.10**	−0.15, −0.04	**<0.001**
(mmol/L)	2	5448	13411	reference	−**0.09**	−0.14, −0.03	−**0.09**	−0.15, −0.04	**<0.001**
	3	5415	13338	reference	−**0.08**	−0.13, −0.02	−**0.06**	−0.11, −0.002	**0.009**
Fasting plasma	1	5806	14384	reference	−0.02	−0.09, 0.06	0.05	−0.03, 0.12	0.34
glucose (mmol/L)	2	5424	13379	reference	0.003	−0.08, 0.08	0.05	−0.03, 0.13	0.24
	3	5394	13306	reference	0.01	−0.07, 0.09	**0.08**	0.01, 0.16	**0.048**
Body mass index	1	5825	14440	reference	−0.10	−0.24, 0.04	−**0.31**	−0.45, −0.17	**<0.001**
	2	5453	13430	reference	−0.07	−0.21, 0.08	−**0.26**	−0.40, −0.11	**<0.001**
Waist-hip ratio	1	5822	14419	reference	−**0.003**	−0.01, −0.001	−**0.01**	−0.01, −0.006	**<0.001**
	2	5442	13409	reference	−0.002	−0.004, 0.001	−**0.01**	−0.01, −0.003	**<0.001**
					OR	95% CI	OR	95% CI	
Diabetes	1	5818	14418	reference	0.98	0.86, 1.12	1.07	0.95, 1.21	0.35
	2	5437	13410	reference	0.98	0.85, 1.12	1.04	0.91, 1.18	0.67
	3	5403	13334	reference	0.99	0.86, 1.13	1.09	0.96, 1.24	0.24

a Censored regression was used for systolic blood pressure, diastolic blood pressure, pulse pressure, HDL, LDL, triglyceride and fasting plasma glucose; linear regression was used for body mass index and waist-hip ratio; logistic regression was used for diabetes.

b Model 1 adjusted for age, sex and phase; Model 2 adjusted for age, sex, phase, SEP (education, father's occupation, longest-held occupation and personal income) and lifestyle (smoking status, alcohol use and physical activity); Model 3 adjusted for age, sex, phase, SEP, lifestyle, BMI and WHR.

c 1 portion = 250 ml.

d Coefficient means changes in risk factors; bold values indicate *P*<0.05.

The associations did not vary with sex (*P*-values >0.14) or age (*P*-values >0.09). However, sex-specific associations are shown in Table S3 in File S1 and Table S4 in File S1. Associations were clearer for women than men.

## Discussion

In this large study from an under-studied non-western developing population, whole cow's milk consumption of more than 3 portions per week was associated with healthier levels of most CVD risk factors considered (systolic blood pressure, diastolic blood pressure, pulse pressure, triglycerides, HDL-cholesterol, BMI and WHR) except fasting glucose and had no obvious associations with LDL-cholesterol or diabetes.

To our knowledge, our study is unique in assessing the associations of whole cow's milk consumption with CVD risk factors in a non-western population whose lifetime experiences are typical of much of the global population. Other strengths include a large sample size and detailed recording of milk consumption frequency and quantity. Nevertheless, limitations exist. First, our study is cross-sectional, so reverse causation is possible. However, after excluding participants with previous CVD or diabetes, results were similar. Second, our participants were not a randomly selected, representative sample. However, sample selection should not affect internal associations, unless we missed people with specific combinations of whole cow's milk consumption and CVD risk factors. Furthermore, associations did not differ by age, which might indicate differential selection into the study. Third, we used recalled consumption of whole cow's milk. Any non-differential misclassification would, most likely, make our results conservative. Systematic recall bias by CVD risk factors is unlikely as participants were unaware of this hypothesis at the time of interview. Fourth, lactase persistence is uncommon in East Asian populations[26]; some Chinese adults might have difficulty digesting cow's milk. However, a study from China suggested that most healthy adults (81.9%) ingested >250 ml cow's milk per portion[27], so our categorization could reflect amounts of cow's milk drunk even by those without lactase persistence. Fifth, BMI and WHR may be mediators rather than confounders of the association of whole milk consumption with fasting plasma glucose as the association was only obvious after adjustment for BMI and WHR. Finally, though we adjusted for life course SEP and lifestyle in our analysis, residual confounding by SEP is still possible. Besides, we were unable to adjust for potential confounding by diet, because of the difficulty of obtaining overall dietary data in large-scale studies of free-living participants[28]. Hence we do not know if the associations with whole cow's milk occurred because cow's milk drinkers had a generally healthier diet. However, after adjusting for soy milk, which is common substitute for cow's milk in China, the results remained similar. Furthermore, how a healthy diet might raise HDL-cholesterol without affecting LDL-cholesterol is unclear.

Our findings of whole cow's milk consumption associated with lower triglycerides and higher HDL-cholesterol, but not with LDL-cholesterol, are consistent with RCTs[14], [15] giving more credence to our other findings, although whole cow's milk was not associated with blood pressure in one small (n = 55) RCT[13]. In meta-analyses of prospective cohort studies from western populations high-fat dairy intake was also not clearly associated with hypertension incidence or blood pressure[29], [30]. To our knowledge, no RCT has reported the effect of whole cow's milk on fasting plasma glucose or type 2 diabetes. Few observational studies from China have assessed the association of milk drinking with CVD risk factors, although one study of middle-aged women found an inverse association of whole cow's milk consumption with type 2 diabetes[20].

Our study could indicate that whole cow's milk consumption is associated with lower risk of CVD but not diabetes, for which potential mechanisms exist. Cow's milk protein contains peptides such as oligopeptides which inhibit angiotensin-converting enzyme, thereby specifically reducing blood pressure[31]. Cow's milk also contains calcium and vitamin D, which may lower blood pressure by acting as a negative regulator of the rennin-angiotensin system and thereby reducing peripheral vascular resistance[32], [33]. However, RCTs have not consistently confirmed protective effects of calcium and/or vitamin D supplementation on blood pressure[34], lipids[35] or CVD[36]. Whole milk contains fatty acids, such as omega-3s which may reduce triglycerides, SBP and DBP[37]. However, it is unclear why whole milk should have heterogeneous associations with CVD risk factors. Few exposures have been observed to have such a pattern; although androgens would be expected from RCTs to have such specific associations with CVD risk factors[38]. Experiments in mice suggest that omega-3s decrease androgens[39]. However, this is highly speculative, as it is unclear whether the omega-3s contained in whole cow's milk are sufficient to have any such effect.

From a public health perspective, our study shows that whole cow's milk consumption was associated with lower risk of some but not all CVD risk factors. Given CVD risk factors do not have the same effect on absolute risk of CVD or ischemic heart disease in China as in the west[40], for reasons which have never been elucidated, policies and recommendation to increase dairy products consumption to prevent CVD should be made with caution, especially in places where there is no local evidence of clear benefits.

## Conclusion

This large study from a non-western, developing country suggests that whole cow's milk consumption has mixed associations with CVD risk factors. However, our results are from an observational study, thus caution should be paid when drawing conclusions. Further research using suitable study designs, such as Mendelian randomization or RCTs, should be implemented to clarify whether the associations of whole cow's milk with CVD risk factors are causal or a reflection of bias and confounding.

## Supporting Information

File S1 Adjusted associations of whole cow's milk consumption with CVD risk factors after multiple imputation in older Chinese (5853 men and 14,482 women), in older Chinese (3729 men and 8849 women) without previous cardiovascular diseases or diabetes, in 5853 older Chinese men, and in 14,482 older Chinese women, separately, in phases and 2 of the Guangzhou Biobank Cohort Study, 2003–2006.
(DOCX)
