# Supplementary material for: Milk Consumption and Cardiovascular Risk Factors in Older Chinese: The Guangzhou Biobank Cohort Study
Source: PLoS One. 2014 Jan 8;9(1):e84813. doi: 10.1371/journal.pone.0084813 (PMC3885601; doi:10.1371/journal.pone.0084813)
Supplement: File S1 — Adjusted associations of whole cow's milk consumption with CVD risk factors after multiple imputation in older Chinese (5853 men and 14,482 women), in older Chinese (3729 men and 8849 women) without previous cardiovascular diseases or diabetes, in 5853 older Chinese men, and in 14,482 older Chinese women, separately, in phases and 2 of the Guangzhou Biobank Cohort Study, 2003–2006. (DOCX) [file pone.0084813.s001.docx]

Table S1 Adjusted Associations of Whole Cow’s Milk Consumption with CVD Risk Factors After Multiple Imputation in Older Chinese (5853 men and 14,482 women) in Phases and 2 of the Guangzhou Biobank Cohort Study, 2003-2006

| ^a^CVD risk factor | ^b^Model |  | | Whole cow’s milk consumption (^c^portions/week) | | | | | | | | | | | | *P*-value  for  trend |
| --- | --- | --- | --- | --- | --- | --- | --- | --- | --- | --- | --- | --- | --- | --- | --- | --- |
|  |  | N | |  | | 0 |  | | 1-3 | | |  | >3 | | |  |
|  |  | Men Women | | |  | | | ^d^coefficient | | | 95% CI | | coefficient | | 95% CI |  |
| Systolic blood | 1 | 5836 | 14448 | | reference | | | **-1.59** | | -2.68, -0.50 | | | **-3.57** | -4.64, -2.50 | | **<0.001** |
| pressure (mm Hg) | 2 | 5836 | 14448 | | reference | | | **-1.38** | | -2.47, -0.29 | | | **-3.23** | -4.31, -2.14 | | **<0.001** |
|  | 3 | 5836 | 14448 | | reference | | | **-1.19** | | -2.24, -0.13 | | | **-2.57** | -3.61, -1.52 | | **<0.001** |
|  |  |  |  | |  | | |  | |  | | |  |  | |  |
| Diastolic blood | 1 | 5835 | 14447 | | reference | | | **-0.86** | | -1.43, -0.30 | | | **-1.83** | -2.38, -1.28 | | **<0.001** |
| pressure (mm Hg) | 2 | 5835 | 14447 | | reference | | | **-0.79** | | -1.36, -0.23 | | | **-1.74** | -2.30, -1.18 | | **<0.001** |
|  | 3 | 5835 | 14447 | | reference | | | **-0.69** | | -1.24, -0.15 | | | **-1.39** | -1.93, -0.86 | | **<0.001** |
|  |  |  |  | |  | | |  | |  | | |  |  | |  |
| Pulse pressure | 1 | 5832 | 14442 | | reference | | | -0.72 | | -1.44, 0.01 | | | **-1.76** | -2.47, -1.05 | | **<0.001** |
| (mm Hg) | 2 | 5832 | 14442 | | reference | | | -0.62 | | -1.34, 0.11 | | | **-1.57** | -2.29, -0.85 | | **<0.001** |
|  | 3 | 5832 | 14442 | | reference | | | -0.52 | | -1.23, 0.20 | | | **-1.23** | -1.93, -0.52 | | **<0.001** |
|  |  |  |  | |  | | |  | |  | | |  |  | |  |
| HDL-cholesterol | 1 | 5838 | 14440 | | reference | | | **0.03** | | 0.01, 0.05 | | | **0.03** | 0.02, 0.05 | | **<0.001** |
| (mmol/L) | 2 | 5838 | 14440 | | reference | | | **0.03** | | 0.01, 0.04 | | | **0.03** | 0.01, 0.04 | | **<0.001** |
|  | 3 | 5838 | 14440 | | reference | | | **0.02** | | 0.01, 0.04 | | | **0.02** | 0.003, 0.04 | | **0.003** |
|  |  |  |  | |  | | |  | |  | | |  |  | |  |
| LDL-cholesterol | 1 | 5824 | 14396 | | reference | | | 0.02 | | -0.01, 0.05 | | | 0.03 | -0.003, 0.05 | | **0.042** |
| (mmol/L) | 2 | 5824 | 14396 | | reference | | | 0.02 | | -0.01, 0.05 | | | 0.02 | -0.01, 0.04 | | 0.19 |
|  | 3 | 5824 | 14396 | | reference | | | 0.02 | | -0.01, 0.05 | | | 0.02 | -0.01, 0.05 | | 0.10 |
|  |  |  |  | |  | | |  | |  | | |  |  | |  |
| Triglycerides | 1 | 5839 | 14445 | | reference | | | **-0.09** | | -0.14, -0.03 | | | **-0.10** | -0.15, -0.04 | | **<0.001** |
| (mmol/L) | 2 | 5839 | 14445 | | reference | | | **-0.08** | | -0.14, -0.03 | | | **-0.09** | -0.15, -0.04 | | **<0.001** |
|  | 3 | 5839 | 14445 | | reference | | | **-0.08** | | -0.13, -0.02 | | | **-0.06** | -0.11, -0.01 | | **0.005** |
|  |  |  |  | |  | | |  | |  | | |  |  | |  |
| Fasting plasma | 1 | 5817 | 14412 | | reference | | | -0.02 | | -0.09, 0.06 | | | 0.05 | -0.03, 0.12 | | 0.34 |
| glucose (mmol/L) | 2 | 5817 | 14412 | | reference | | | -0.004 | | -0.08, 0.07 | | | 0.06 | -0.01, 0.14 | | 0.15 |
|  | 3 | 5817 | 14412 | | reference | | | 0.004 | | -0.07, 0.08 | | | **0.10** | 0.02, 0.17 | | **0.023** |
|  |  |  |  | |  | | |  | |  | | |  |  | |  |
| Body mass index | 1 | 5835 | 14440 | | reference | | | -0.10 | | -0.24, 0.04 | | | **-0.31** | -0.45, -0.17 | | **<0.001** |
|  | 2 | 5835 | 14440 | | reference | | | -0.07 | | -0.21, 0.07 | | | **-0.27** | -0.40, -0.13 | | **<0.001** |
|  |  |  |  | |  | | |  | |  | | |  |  | |  |
| Waist-hip ratio | 1 | 5822 | 14419 | | reference | | | **-0.003** | | -0.01, -0.005 | | | **-0.01** | -0.01, -0.006 | | **<0.001** |
|  | 2 | 5822 | 14419 | | reference | | | -0.001 | | -0.004, 0.001 | | | **-0.01** | -0.01, -0.003 | | **<0.001** |
|  |  |  |  | |  | | | OR | | 95% CI | | | OR | 95% CI | |  |
| Diabetes | 1 | 5818 | 14418 | | reference | | | 0.98 | | 0.86, 1.12 | | | 1.07 | 0.95, 1.21 | | 0.35 |
|  | 2 | 5818 | 14418 | | reference | | | 0.98 | | 0.86, 1.12 | | | 1.06 | 0.94, 1.20 | | 0.41 |
|  | 3 | 5818 | 14418 | | reference | | | 0.99 | | 0.87, 1.13 | | | 1.12 | 0.99, 1.27 | | 0.12 |

^a^Censored regression was used for systolic blood pressure, diastolic blood pressure, pulse pressure, HDL, LDL, triglyceride and fasting plasma glucose; linear regression was used for body mass index and waist-hip ratio; logistic regression was used for diabetes.

^b^Model 1 adjusted for age, sex and phase; Model 2 adjusted for age, sex, phase, SEP (education, father’s occupation, longest-held occupation and personal income) and lifestyle (smoking status, alcohol use and physical activity); Model 3 adjusted for age, sex, phase, SEP, lifestyle, BMI and WHR.

^c^1 portion=250ml.

^d^Coefficient means changes in risk factors; bold values indicate *P* <0.05.

Table S2 Adjusted Associations of Whole Cow’s Milk Consumption with CVD Risk Factors in Older Chinese (3729 men and 8849 women) without Previous Cardiovascular Diseases or Diabetes in Phases 1 and 2 of the Guangzhou Biobank Cohort Study, 2003-2006

| ^a^CVD risk factor | ^b^Model |  | | | Whole cow’s milk consumption (^c^portions/week) | | | | | | | | | | | *P*-value  for trend |
| --- | --- | --- | --- | --- | --- | --- | --- | --- | --- | --- | --- | --- | --- | --- | --- | --- |
|  |  | N | |  | | 0 |  | | 1-3 | | |  | >3 | | |  |
|  |  | Men Women | | |  | | | ^d^coefficient | | | 95% CI | | coefficient | | 95% CI |  |
| Systolic blood | 1 | 3728 | 8846 | | reference | | | **-1.89** | | -2.91, -0.87 | | | **-3.18** | -4.18, -2.18 | | **<0.001** |
| pressure (mm Hg) | 2 | 3470 | 8155 | | reference | | | **-1.36** | | -2.40, -0.31 | | | **-2.44** | -3.47, -1.41 | | **<0.001** |
|  | 3 | 3456 | 8123 | | reference | | | **-1.15** | | -2.17, -0.13 | | | **-2.04** | -3.05, -1.03 | | **<0.001** |
|  |  |  |  | |  | | |  | |  | | |  |  | |  |
| Diastolic blood | 1 | 3727 | 8845 | | reference | | | **-1.08** | | -1.63, -0.54 | | | **-1.62** | -2.16, -1.09 | | **<0.001** |
| pressure (mm Hg) | 2 | 3469 | 8154 | | reference | | | **-0.86** | | -1.42, -0.29 | | | **-1.27** | -1.83, -0.72 | | **<0.001** |
|  | 3 | 3455 | 8122 | | reference | | | **-0.73** | | -1.27, -0.18 | | | **-1.04** | -1.58, -0.50 | | **<0.001** |
|  |  |  |  | |  | | |  | |  | | |  |  | |  |
| Pulse pressure | 1 | 3726 | 8844 | | reference | | | **-0.80** | | -1.48, -0.13 | | | **-1.56** | -2.22, -0.89 | | **<0.001** |
| (mm Hg) | 2 | 3468 | 8153 | | reference | | | -0.50 | | -1.19, 0.20 | | | **-1.18** | -1.87, -0.49 | | **<0.001** |
|  | 3 | 3454 | 8121 | | reference | | | -0.42 | | -1.11, 0.28 | | | **-1.01** | -1.69, -0.32 | | **0.003** |
|  |  |  |  | |  | | |  | |  | | |  |  | |  |
| HDL-cholesterol | 1 | 3701 | 8809 | | reference | | | **0.04** | | 0.02, 0.06 | | | **0.04** | 0.02, 0.06 | | **<0.001** |
| (mmol/L) | 2 | 3444 | 8120 | | reference | | | **0.03** | | 0.01, 0.06 | | | **0.03** | 0.01, 0.05 | | **0.0014** |
|  | 3 | 3430 | 8087 | | reference | | | **0.03** | | 0.01, 0.05 | | | **0.02** | 0.01, 0.04 | | **0.0095** |
|  |  |  |  | |  | | |  | |  | | |  |  | |  |
| LDL-cholesterol | 1 | 3718 | 8825 | | reference | | | 0.02 | | -0.02, 0.05 | | | **0.04** | 0.002, 0.07 | | **0.029** |
| (mmol/L) | 2 | 3461 | 8135 | | reference | | | 0.01 | | -0.02, 0.05 | | | 0.03 | -0.01, 0.06 | | 0.11 |
|  | 3 | 3447 | 8102 | | reference | | | 0.02 | | -0.02, 0.05 | | | 0.03 | -0.004, 0.07 | | 0.074 |
|  |  |  |  | |  | | |  | |  | | |  |  | |  |
| Triglycerides | 1 | 3708 | 8788 | | reference | | | **-0.09** | | -0.15, -0.04 | | | **-0.08** | -0.14, -0.02 | | **<0.001** |
| (mmol/L) | 2 | 3451 | 8100 | | reference | | | **-0.08** | | -0.14, -0.02 | | | -0.05 | -0.11, 0.01 | | **0.017** |
|  | 3 | 3437 | 8067 | | reference | | | **-0.07** | | -0.13, -0.01 | | | -0.03 | -0.09, 0.03 | | 0.13 |
|  |  |  |  | |  | | |  | |  | | |  |  | |  |
| Fasting plasma | 1 | 3717 | 8823 | | reference | | | -0.06 | | -0.13, 0.001 | | | -0.05 | -0.12, 0.01 | | **0.038** |
| glucose (mmol/L) | 2 | 3460 | 8133 | | reference | | | -0.04 | | -0.11, 0.03 | | | -0.04 | -0.11, 0.03 | | 0.15 |
|  | 3 | 3446 | 8100 | | reference | | | -0.03 | | -0.10, 0.04 | | | -0.02 | -0.09, 0.05 | | 0.41 |
|  |  |  |  | |  | | |  | |  | | |  |  | |  |
| Body mass index | 1 | 3726 | 8838 | | reference | | | -0.15 | | -0.32, 0.02 | | | **-0.27** | -0.44, -0.11 | | **<0.001** |
|  | 2 | 3468 | 8147 | | reference | | | -0.11 | | -0.29, 0.07 | | | **-0.22** | -0.39, -0.04 | | **0.011** |
|  |  |  |  | |  | | |  | |  | | |  |  | |  |
| Waist-hip ratio | 1 | 3716 | 8824 | | reference | | | **-0.004** | | -0.01, -0.001 | | | **-0.01** | -0.01, -0.005 | | **<0.001** |
|  | 2 | 3460 | 8132 | | reference | | | -0.003 | | -0.01, 0.001 | | | **-0.01** | -0.01, -0.002 | | **<0.001** |
|  |  |  |  | |  | | | OR | | 95% CI | | | OR | 95% CI | |  |
| Diabetes | 1 | 3701 | 8809 | | reference | | | 0.**75** | | 0.56, 0.99 | | | 0.85 | 0.64, 1.10 | | 0.087 |
|  | 2 | 3444 | 8120 | | reference | | | 0.76 | | 0.55, 1.03 | | | 0.87 | 0.65, 1.15 | | 0.17 |
|  | 3 | 3430 | 8087 | | reference | | | 0.78 | | 0.57, 1.05 | | | 0.93 | 0.69, 1.23 | | 0.34 |

^a^Linear regression was used for systolic blood pressure, diastolic blood pressure, pulse pressure, HDL, LDL, triglyceride, fasting plasma glucose, body mass index and waist-hip ratio; logistic regression was used for diabetes.

^b^Model 1 adjusted for age, sex and phase; Model 2 adjusted for age, sex, phase, SEP (education, father’s occupation, longest-held occupation and personal income) and lifestyle (smoking status, alcohol use and physical activity); Model 3 adjusted for age, sex, phase, SEP, lifestyle, BMI and WHR.

^c^1 portion=250ml.

^d^Coefficient means changes in risk factors; bold values indicate *P*<0.05.

Table S3 Adjusted Associations of Whole Cow’s Milk Consumption with CVD Risk Factors in 5853 Older Chinese Men in Phases 1 and 2 of the Guangzhou Biobank Cohort Study, 2003-2006

| ^a^CVD risk factor | ^b^Model | N | Whole cow’s milk consumption (^c^portions/week) | | | | | | | | | | | *P*-value  for trend |
| --- | --- | --- | --- | --- | --- | --- | --- | --- | --- | --- | --- | --- | --- | --- |
|  |  |  | 0 |  | | 1-3 | | |  | | >3 | | |  |
|  |  |  |  | | ^d^coefficient | | | 95% CI | | coefficient | | | 95% CI |  |
| Systolic blood | 1 | 5826 | reference | | -0.95 | | -3.00, 1.09 | | | **-4.08** | | -6.04, -2.13 | | **<0.001** |
| pressure (mm Hg) | 2 | 5445 | reference | | -1.36 | | -3.44, 0.72 | | | **-4.64** | | -6.66, -2.62 | | **<0.001** |
|  | 3 | 5424 | reference | | -0.96 | | -2.94, 1.01 | | | **-3.97** | | -5.89, -2.05 | | **<0.001** |
|  |  |  |  | |  | |  | | |  | |  | |  |
| Diastolic blood | 1 | 5825 | reference | | -0.56 | | -1.65, 0.53 | | | **-2.24** | | -3.29, -1.20 | | **<0.001** |
| pressure (mm Hg) | 2 | 5444 | reference | | -0.77 | | -1.88, 0.35 | | | **-2.52** | | -3.60, -1.44 | | **<0.001** |
|  | 3 | 5423 | reference | | -0.56 | | -1.61, 0.49 | | | **-2.16** | | -3.18, -1.14 | | **<0.001** |
|  |  |  |  | |  | |  | | |  | |  | |  |
| Pulse pressure | 1 | 5822 | reference | | -0.31 | | -1.64, 1.03 | | | **-1.90** | | -3.18, -0.62 | | **0.0056** |
| (mm Hg) | 2 | 5441 | reference | | -0.62 | | -1.99, 0.76 | | | **-2.30** | | -3.63, -0.97 | | **<0.001** |
|  | 3 | 5420 | reference | | -0.41 | | -1.75, 0.93 | | | **-1.97** | | -3.27, -0.67 | | **0.0044** |
|  |  |  |  | |  | |  | | |  | |  | |  |
| HDL-cholesterol | 1 | 5826 | reference | | 0.03 | | -0.001, 0.06 | | | 0.02 | | -0.01, 0.05 | | 0.090 |
| (mmol/L) | 2 | 5447 | reference | | **0.04** | | 0.01, 0.07 | | | 0.02 | | -0.01, 0.05 | | 0.067 |
|  | 3 | 5414 | reference | | **0.04** | | 0.003, 0.07 | | | 0.02 | | -0.02, 0.05 | | 0.14 |
|  |  |  |  | |  | |  | | |  | |  | |  |
| LDL-cholesterol | 1 | 5812 | reference | | 0.02 | | -0.03, 0.07 | | | -0.003 | | -0.05, 0.05 | | 0.92 |
| (mmol/L) | 2 | 5433 | reference | | 0.02 | | -0.03, 0.08 | | | -0.0001 | | -0.05, 0.05 | | 0.82 |
|  | 3 | 5400 | reference | | 0.03 | | -0.02, 0.08 | | | 0.01 | | -0.04, 0.06 | | 0.53 |
|  |  |  |  | |  | |  | | |  | |  | |  |
| Triglycerides | 1 | 5827 | reference | | -0.07 | | -0.17, 0.04 | | | -0.09 | | -0.19, 0.01 | | **0.045** |
| (mmol/L) | 2 | 5448 | reference | | -0.09 | | -0.19, 0.02 | | | **-0.12** | | -0.22, -0.02 | | **0.011** |
|  | 3 | 5415 | reference | | -0.08 | | -0.18, 0.03 | | | -0.09 | | -0.19, 0.01 | | **0.044** |
|  |  |  |  | |  | |  | | |  | |  | |  |
| Fasting plasma | 1 | 5806 | reference | | 0.04 | | -0.09, 0.18 | | | 0.09 | | -0.04, 0.22 | | 0.16 |
| glucose (mmol/L) | 2 | 5424 | reference | | 0.01 | | -0.13, 0.15 | | | 0.04 | | -0.10, 0.17 | | 0.60 |
|  | 3 | 5394 | reference | | 0.02 | | -0.12, 0.16 | | | 0.06 | | -0.07, 0.20 | | 0.36 |
|  |  |  |  | |  | |  | | |  | |  | |  |
| Body mass index | 1 | 5825 | reference | | -0.10 | | -0.36, 0.16 | | | -0.13 | | -0.37, 0.12 | | 0.26 |
|  | 2 | 5453 | reference | | -0.17 | | -0.44, 0.10 | | | -0.23 | | -0.49, 0.03 | | **0.048** |
|  |  |  |  | |  | |  | | |  | |  | |  |
| Waist-hip ratio | 1 | 5822 | reference | | -0.001 | | -0.01, 0.004 | | | -0.004 | | -0.01, 0.001 | | 0.15 |
|  | 2 | 5442 | reference | | -0.001 | | -0.01, 0.004 | | | -0.01 | | -0.01, 0.0004 | | 0.079 |
|  |  |  |  | | OR | | 95% CI | | | OR | | 95% CI | |  |
| Diabetes | 1 | 5818 | reference | | 1.00 | | 0.77, 1.28 | | | 1.15 | | 0.91, 1.44 | | 0.29 |
|  | 2 | 5437 | reference | | 0.92 | | 0.70, 1.19 | | | 0.99 | | 0.77, 1.25 | | 0.78 |
|  | 3 | 5403 | reference | | 0.93 | | 0.71, 1.21 | | | 1.03 | | 0.80, 1.31 | | 0.97 |

^a^Censored regression was used for systolic blood pressure, diastolic blood pressure, pulse pressure, HDL, LDL, triglyceride and fasting plasma glucose; linear regression was used for body mass index and waist-hip ratio; logistic regression was used for diabetes.

^b^Model 1 adjusted for age and phase; Model 2 adjusted for age, phase, SEP (education, father’s occupation, longest-held occupation and personal income) and lifestyle (smoking status, alcohol use and physical activity); Model 3 adjusted for age,, phase, SEP, lifestyle, BMI and WHR.

^c^1 portion=250ml.

^d^Coefficient means changes in risk factors; bold values indicate *P*<0.05.

Table S4 Adjusted Associations of Whole Cow’s Milk Consumption with CVD Risk Factors in 14,482 Older Chinese Women in Phases 1 and 2 of the Guangzhou Biobank Cohort Study, 2003-2006

| ^a^CVD risk factor | ^b^Model | N | Whole cow’s milk consumption (^c^portions/week) | | | | | | | | | | | *P*-value  for trend |
| --- | --- | --- | --- | --- | --- | --- | --- | --- | --- | --- | --- | --- | --- | --- |
|  |  |  | 0 |  | | 1-3 | | |  | | >3 | | |  |
|  |  |  |  | | ^d^coefficient | | | 95% CI | | coefficient | | | 95% CI |  |
| Systolic blood | 1 | 14431 | reference | | **-1.78** | | -3.07, -0.49 | | | **-3.31** | | -4.59, -2.03 | | **<0.001** |
| pressure (mm Hg) | 2 | 13422 | reference | | -1.31 | | -2.65, 0.03 | | | **-2.73** | | -4.05, -1.41 | | **<0.001** |
|  | 3 | 13369 | reference | | -1.11 | | -2.41, 0.19 | | | **-1.97** | | -3.26, -0.68 | | **0.0014** |
|  |  |  |  | |  | |  | | |  | |  | |  |
| Diastolic blood | 1 | 14430 | reference | | **-0.95** | | -1.61, -0.30 | | | **-1.65** | | -2.30, -1.00 | | **<0.001** |
| pressure (mm Hg) | 2 | 13421 | reference | | **-0.72** | | -1.40, -0.03 | | | **-1.36** | | -2.04, -0.69 | | **<0.001** |
|  | 3 | 13368 | reference | | -0.60 | | -1.26, 0.06 | | | **-0.96** | | -1.61, -0.30 | | **0.0019** |
|  |  |  |  | |  | |  | | |  | |  | |  |
| Pulse pressure | 1 | 14425 | reference | | -0.85 | | -1.72, 0.02 | | | **-1.67** | | -2.52, -0.81 | | **<0.001** |
| (mm Hg) | 2 | 13416 | reference | | -0.66 | | -1.56, 0.23 | | | **-1.44** | | -2.32, -0.55 | | **0.0010** |
|  | 3 | 13363 | reference | | -0.57 | | -1.46, 0.31 | | | **-1.04** | | -1.92, -0.16 | | **0.014** |
|  |  |  |  | |  | |  | | |  | |  | |  |
| HDL-cholesterol | 1 | 14414 | reference | | **0.03** | | 0.01, 0.05 | | | **0.04** | | 0.02, 0.06 | | **<0.001** |
| (mmol/L) | 2 | 13407 | reference | | **0.02** | | 0.002, 0.05 | | | **0.03** | | 0.01, 0.05 | | **<0.001** |
|  | 3 | 13334 | reference | | 0.02 | | -0.001, 0.04 | | | **0.03** | | 0.01, 0.05 | | **0.0051** |
|  |  |  |  | |  | |  | | |  | |  | |  |
| LDL-cholesterol | 1 | 14370 | reference | | 0.02 | | -0.01, 0.06 | | | **0.04** | | 0.003, 0.07 | | **0.021** |
| (mmol/L) | 2 | 13364 | reference | | 0.01 | | -0.02, 0.05 | | | 0.03 | | -0.01, 0.06 | | 0.15 |
|  | 3 | 13291 | reference | | 0.02 | | -0.02, 0.05 | | | 0.03 | | -0.01, 0.07 | | 0.094 |
|  |  |  |  | |  | |  | | |  | |  | |  |
| Triglycerides | 1 | 14419 | reference | | **-0.09** | | -0.16, -0.03 | | | **-0.10** | | -0.16, -0.04 | | **<0.001** |
| (mmol/L) | 2 | 13411 | reference | | **-0.08** | | -0.15, -0.01 | | | **-0.08** | | -0.15, -0.02 | | **0.0037** |
|  | 3 | 13338 | reference | | **-0.07** | | -0.14, -0.01 | | | -0.05 | | -0.11, 0.02 | | 0.058 |
|  |  |  |  | |  | |  | | |  | |  | |  |
| Fasting plasma | 1 | 14384 | reference | | -0.04 | | -0.13, 0.05 | | | 0.03 | | -0.06, 0.12 | | 0.74 |
| glucose (mmol/L) | 2 | 13379 | reference | | -0.01 | | -0.10, 0.09 | | | 0.06 | | -0.04, 0.15 | | 0.32 |
|  | 3 | 13306 | reference | | 0.01 | | -0.09, 0.10 | | | 0.09 | | -0.002, 0.19 | | 0.078 |
|  |  |  |  | |  | |  | | |  | |  | |  |
| Body mass index | 1 | 14440 | reference | | -0.11 | | -0.27, 0.06 | | | **-0.38** | | -0.54, -0.22 | | **<0.001** |
|  | 2 | 13430 | reference | | -0.03 | | -0.21, 0.14 | | | **-0.27** | | -0.45, -0.10 | | **0.0035** |
|  |  |  |  | |  | |  | | |  | |  | |  |
| Waist-hip ratio | 1 | 14419 | reference | | **-0.004** | | -0.01, -0.001 | | | **-0.01** | | -0.01, -0.007 | | **<0.001** |
|  | 2 | 13409 | reference | | -0.002 | | -0.01, 0.001 | | | **-0.01** | | -0.01, -0.004 | | **<0.001** |
|  |  |  |  | | OR | | 95% CI | | | OR | | 95% CI | |  |
| Diabetes | 1 | 14418 | reference | | 0.98 | | 0.84, 1.14 | | | 1.05 | | 0.91, 1.20 | | 0.62 |
|  | 2 | 13410 | reference | | 1.00 | | 0.85, 1.16 | | | 1.06 | | 0.91, 1.23 | | 0.49 |
|  | 3 | 13334 | reference | | 1.01 | | 0.86, 1.18 | | | 1.12 | | 0.97, 1.31 | | 0.16 |

^a^Censored regression was used for systolic blood pressure, diastolic blood pressure, pulse pressure, HDL, LDL, triglyceride and fasting plasma glucose; linear regression was used for body mass index and waist-hip ratio; logistic regression was used for diabetes.

^b^Model 1 adjusted for age and phase; Model 2 adjusted for age, phase, SEP (education, father’s occupation, longest-held occupation and personal income) and lifestyle (smoking status, alcohol use and physical activity); Model 3 adjusted for age, phase, SEP, lifestyle, BMI and WHR.

^c^1 portion=250ml.

^d^Coefficient means changes in risk factors; bold values indicate *P*<0.05.
